# Artifact Noise Removal Techniques on Seismocardiogram Using Two Tri-Axial Accelerometers

**DOI:** 10.3390/s18041067

**Published:** 2018-04-02

**Authors:** Loc Luu, Anh Dinh

**Affiliations:** Department of Electrical and Computer Engineering, University of Saskatchewan, Saskatoon, SK S7N 5A9, Canada; loc.luu@usask.ca

**Keywords:** artifact noise removal, seismocardiogram, accelerometers, sensor locations

## Abstract

The aim of this study is on the investigation of motion noise removal techniques using two-accelerometer sensor system and various placements of the sensors on gentle movement and walking of the patients. A Wi-Fi based data acquisition system and a framework on Matlab are developed to collect and process data while the subjects are in motion. The tests include eight volunteers who have no record of heart disease. The walking and running data on the subjects are analyzed to find the minimal-noise bandwidth of the SCG signal. This bandwidth is used to design filters in the motion noise removal techniques and peak signal detection. There are two main techniques of combining signals from the two sensors to mitigate the motion artifact: analog processing and digital processing. The analog processing comprises analog circuits performing adding or subtracting functions and bandpass filter to remove artifact noises before entering the data acquisition system. The digital processing processes all the data using combinations of total acceleration and z-axis only acceleration. The two techniques are tested on three placements of accelerometer sensors including horizontal, vertical, and diagonal on gentle motion and walking. In general, the total acceleration and z-axis acceleration are the best techniques to deal with gentle motion on all sensor placements which improve average systolic signal-noise-ratio (SNR) around 2 times and average diastolic SNR around 3 times comparing to traditional methods using only one accelerometer. With walking motion, ADDER and z-axis acceleration are the best techniques on all placements of the sensors on the body which enhance about 7 times of average systolic SNR and about 11 times of average diastolic SNR comparing to only one accelerometer method. Among the sensor placements, the performance of horizontal placement of the sensors is outstanding comparing with other positions on all motions.

## 1. Introduction

Understanding the heart activities in every cardiac cycle is very useful in evaluating its performance. Monitoring the heart activities is extremely useful in screening and detecting of many cardiac related diseases. With the advance of technology, seismocardiography (SCG) observed by Bozhenko in 1962 [1] is easier to record compared to ballistocardiogram (BCG) and SCG applications in healthcare become reality. The SCG signal is sensed by an accelerometer placed on the chest or other parts of a body. SCG has been used to diagnose coronary artery diseases two decades ago [2]. Compared to ECG, SCG was proven to have a better sensitivity for identifying anatomic and physiologic ischemic coronary artery disease during exercise stress testing on women and on patients who did not reach the maximum heart beat in the test. Moreover, the technique also provides more information for diagnosis than exercise ECG [2].

The authors in [3] suggested the benefits of SCG when observing the left ventricular function during myocardial ischemia. In [4], the change of SCG before and after balloon angioplasty was also analyzed to prove that SCG varied due to ischemia changes caused by decreased coronary blood flow [4]. The authors in [5] measured the left ventricular functions on 58 subjects using SCG and compared with literature; the SCG provided good data for all functions even with exercise while literature did not. The cardiac time intervals measured by SCG and ECG were studied on two groups of 12 women running on treadmill to determine the difference between trained and untrained women [6]. Doctors in [7] investigated the systolic time intervals (STIs) with left ventricular ejection time (LVET) and pre-ejection period (PEP) extracted from an accelerometer on the chest of young patients in head upright and tilt testing. In some special cases, the patients were required to monitor cardiac function under stress test with MRI. However, the ECG was not compatible for cardiac function with MRI due to the distortion of high electrical voltages caused by MRI. In contrast, SCG was experimented on animal in the same configuration to prove SCG as a solution to detect myocardial ischemia during MRI [8]. Recent works on the applications of SCG to cardiac performance have been published [9,10,11]. Authors in [12] uses a 6-degree of freedom inertia sensor module to estimate the heart rate from SCG.

**Figure 1:**
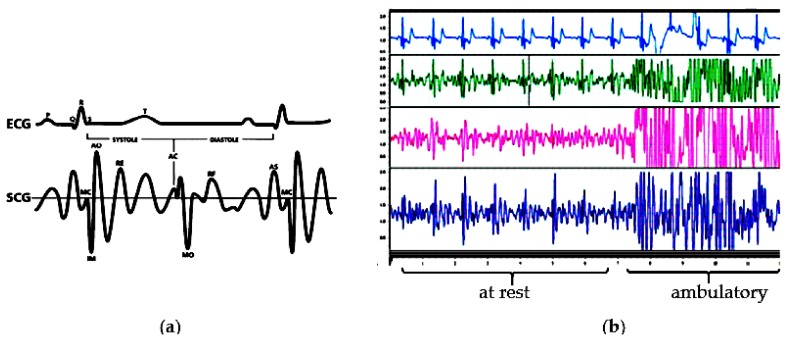
(**a**) Example of ECG and SCG beats simultaneously recorded [2]. (**b**) ECG, x, y, and z axis of the SCG during resting and walking from experimental results.

Figure 1a shows the peaks of the SCG waveform corresponding to the R peaks of the ECG. As shown, heart rate can be easily calculated from the SCG. The derivation of the heart rate from the accelerometer based system works well when the patient is at rest, either sitting, standing or laying down. However, when the patient is on the move (i.e., ambulatory), the motion artifact picked up by the accelerometer is much stronger than the heart motion signals received at the chest wall. These strong signals destroy the SCG waves from the heart [13,14]. Figure 1b shows the display of the 3 axes (x, y, z) of the acceleration sensor when the body is at rest and when the body is in motion in which the z-axis is pointing inward to the chest. As seen, the SCG signals are not useful due to the corruption from the motion artifact. Obviously, it is very difficult to extract the heart rate or identify the peaks in the SCG waveform collected from the accelerometer signals under ambulatory conditions. Simple methods must be sought to reduce motion artifact in order to record accurate SCG in all environment. There are a number of methods and techniques in motion artifact reduction but they are mostly for photoplethysmography (PPG) and electrocardiography [15,16].

Motion artifact removal of SCG signals has not been thoroughly studied. A study analyzed, identified and eliminated the segments of SCG signal containing some kind of noise which was created at a specific time during recoding [17]. The authors did not describe the method to eliminate the noise beyond an analog band-pass filter before going to the analog-to-digital converter. In another work, various types of noise affecting to SCG signal in different situations were only analyzed without the methods to remove the noise [18]. The article did not show any method to reduce the noise rather than a digital band-pass filter in the pre-processing stage. Although some techniques were applied to remove the motion artifact of SCG signals, they were still complicated and not fully conducted in different types of motion noise [19,20]. The authors in [19] used the normalized least mean square adaptive filter to reduce the motion artifact caused by walking around 1.3 m/s. In [20], empirical mode decomposition was used to denoise walking motion on SCG. Other research works have been recently published investigate different methods in attempting to reduce the effect of motion noise on SCG signal [21,22,23].

All the above studies used only one accelerometer placing on the sternum. Recently, multi-site and multi-location accelerometer concepts on SCG have been investigated and reported in [24,25]. In addition, artifact noise removal techniques have been deployed widely in many areas but the use of a second accelerator to mitigate artifact noise in SCG is the first attempt. This work investigated motion noise removal methods using a system of two accelerometers placed in various positions on the chest. The study will determine which senor placements can achieve the high quality SCG signal while the subjects are in motion in different scenarios including moving gently and walking. In addition, the combination of all three axes of the accelerometer is also to be examined to see if a better detection can be obtained rather than using only z-axis as in many previous studies. The work shows that the use of the multi-accelerometer concept to reduce artifact noise has better performance compared with the single sensor method for the price of cost and complexity.

## 2. Methodology

### 2.1. Wireless Data Acquisition System, Sensing System and Testing Subjects

**Figure 2:**
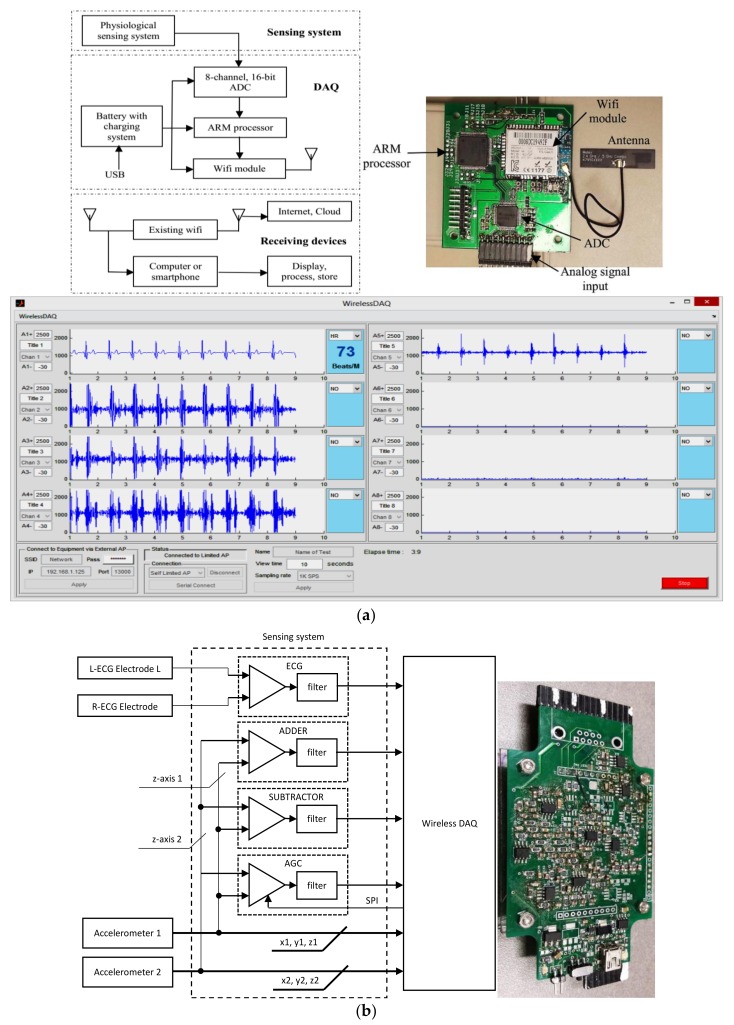
(**a**) Data acquisition system, top-down, clockwise. System block diagram. Photograph of the wireless DAQ (power supply and rechargeable battery are not shown). Screen capture of the DAQ user interface on the computer, (**b**) Block diagram and photograph of the sensing system.

The DAQ system consists of a rechargeable power supply, a 16-bit, 8-channel analog to digital converter, an ARM microcontroller, and a Wi-Fi module packed in a single PCB. The ADC is sampled at a rate of 1000 sample per second. Figure 2 shows the block diagram of the designed system including sensing front-ends, wireless DAQ and the receiving devices. Figure 2 also illustrates the receiving interface of the wireless DAQ on a computer in which custom software was developed to capture, display, analyze, and store the data. In the sensing system, different sensors were used to capture the SCG and ECG signals. They are a pair of high sensitive accelerometers and non-contact dry ECG electrodes. The usage of instrumentation amplifier and op-amp are for noise removal, amplification and filtration.

Figure 2 also illustrates a diagram of the sensing system and its photograph. To collect ECG signal, two non-contact sensors PS25255 (Plessey Semiconductor) were attached horizontally on the subject chest to capture the modified lead-I ECG. The signals from the two ECG sensors were then amplified and filtered (5–50 Hz) using instrumentation amplifier and op-amp. The accelerometers used in the system were made by Kionix, part number KXR94-2050-RF. This ±2 g MEMS device provides 3 analog outputs for x,y,z accelerations with a sensitivity of 660 mV/g. The accelerometer has an operating bandwidth between 0–50 Hz on a 3.3 V power supply. These two tri-axial accelerometers were stacked above the two ECG sensors. The accelerometer on the ECG positive electrode obtained strong SCG signal with motion noise (ACC_SCG) while the accelerometer on the ECG negative electrode collected motion noise and a very weak SCG signal (ACC_MOV).

SCG and ECG were simultaneously recorded on eight volunteers who have no record relating to heart disease with age 32.1 ± 4.5 years, weight 78.2 ± 12.7 kg and height 171.1 ± 7.5 cm. Each subject performed one minute for stationary, one minute for sitting on a chair and moving back and forth gently, one minute for walking on a treadmill with a speed around 5 km/h (around 80 m/s) and one minute for running on treadmill with a speed approximate 8 km/h (around 130 m/s).

### 2.2. Placement of 2 Sensors

**Figure 3:**
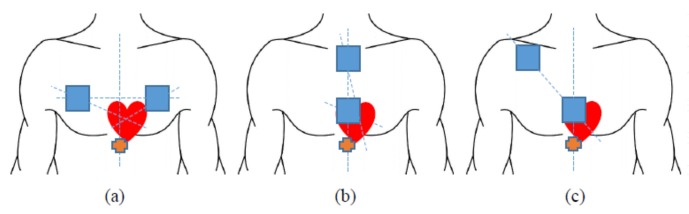
Sensor placements. (**a**) on the same horizontal level. (**b**) on the same vertical line. (**c**) on the diagonal line.

The purpose in this study is to measure the SCG signal on active people so it needs to deal with the body movement noise and the use of two sensors (accelerometers) placed in different positions on the chest is investigated. It is assumed that one sensor is placed near to the heart to capture strong SCG signal with body movement and another one is placed far from the heart to capture body movement with minimized effect of the heart vibration. By observation, the noticed strongest SCG signal is below the chest which is indicated by 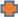 in Figure 3, but it was decided not to put the sensors there because the signals were highly affected by not only body the movement but also respiration.

To minimize the effect of respiration, the sensors were placed on the chest bones. The positions of sensors are 3 types: the same horizontal level on chest wall, the same vertical line on middle of chest wall and on diagonal line goes through the heart on chest wall as illustrated in Figure 3. The activities will also be divided into two categories: gentle movement and walking.

### 2.3. Motion Noise Removal Techniques

A variety of techniques was applied to eliminate the motion noise on SCG using two accelerometers with different placements. For comparison purpose, the simple motion noise removal technique using only one accelerometer with an analog band-pass filter was also conducted. All techniques using two accelerometers can be classified in two main categories of only analog processing and only digital processing. All motion removal methods measured SCG signal along with ECG signal as reference for analysis purpose.

**Figure 4:**
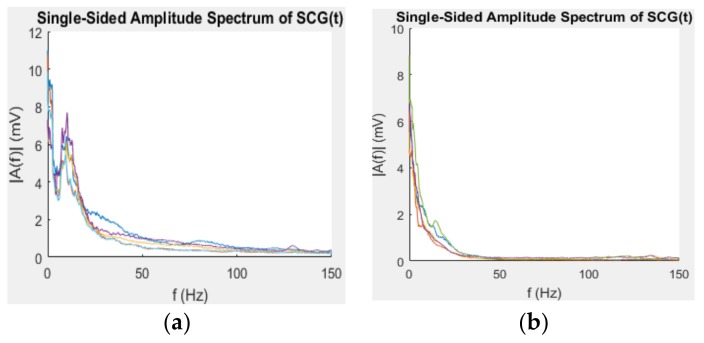
Spectrum of five subjects with (**a**) running and (**b**) walking.

First, the SCG bandwidth is analyzed as the noise frequency is the main drive in noise reduction methods. Two sensors were positioned on the chest of the testing subject, one is placed near to the heart to capture SCG signal and motion, and the other one is placed far from the heart to capture motion and a much smaller SCG signal compared to the other sensor. The signal of the sensors placing near to the heart of five subjects were selected and analyzed using fast fourier transform to identify the high-energy range of the artificial noise including respiratory, body movement (running and walking) and voice. In this work, the noise from voice is ignored. These noises are picked up by the accelerometers in addition to the clear SCG signal. The respiratory rate is in the range of 8–44 per minute corresponding to 0.13–0.73 Hz [26]. The signal picked up by an accelerometer has a range of 0–50 Hz. Figure 4 shows that the motion energy concentrates in the range from 0 to 20 Hz. The frequency of SCG spreads from 0 to 50 Hz [27]; as a result, the effective frequency range was chosen from 20 to 50 Hz.

#### 2.3.1. Motion Noise Removal Using One Accelerometer

**Figure 5:**
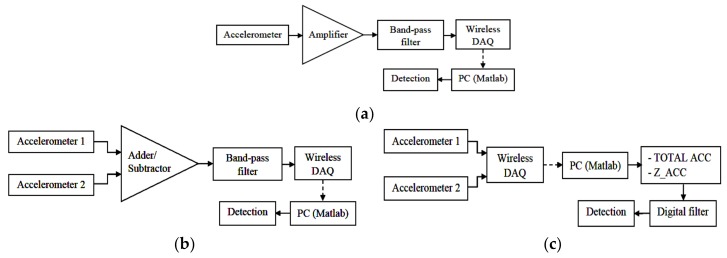
Block diagram (**a**) noise removal using one accelerometer for comparison, (**b**) analog processing steps, and (**c**) digital processing steps.

Many studies used only z-axis of one accelerometer with a filter to measure the SCG signal, in this study, the same configuration on an active person was used to exam the effect of motion noise on the measured SCG signal. The accelerometer was attached on the sternum to obtaining the SCG signal. The results of this configuration were also used to compare the performance with the proposed techniques using two accelerometers. Figure 5a depicts the overview of the simple method using one accelerometer and the band-pass filter and implemented in this work for comparison only. In order to achieve good SCG signal, the total amplifier gain of the band-pass filter was set to around 480. The analog band-pass filter was configured as a second-order with a cut-off frequency between 20 Hz and 50 Hz. The output signal has an offset of 1.25 V and inversion because the amplifier is configured as negative feedback, the signal is to be inverted in the Matlab framework.

#### 2.3.2. Analog Signal Processing on 2 Accelerometers

The analog processing includes an ADDER and a SUBTRACTOR set-up from an op-amp and an instrumentation amplifier as shown in Figure 5b. This configuration removes movement noise by combining two signals from a pair of accelerometers placed at different positions; then, the output signal goes through an analog second-order band-pass filter with a bandwidth of 20 Hz to 50 Hz. The filtered signal is then entered the DAQ to go through the detection algorithms. Due to the complexity of hardware configuration, only the z-axis of the two accelerometers are used for ADDER and SUBTRACTOR. The diagram in Figure 5b illustrates the analog processing.

A real ADDER and SUBTRACTOR with the same placement of sensors for each type (horizontal, vertical and diagonal) were also tested on a subject made gentle movement and walking. There are two important notices when placing the accelerometers on the chest. To eliminate the motion noise from both accelerometers by summing their z-axes, both sensors z-axis must be placed in opposite directions. For example, one sensor has z-axis faces outward from the chest, and another must be placed with the z-axis inward to the chest. With the SUBTRACTOR, since the common-mode rejection of the instrumentation amplifier is used to remove the motion noise, the two z-axes of the accelerometers need to be placed in the same direction. The ADDER uses the MCP6L02 op-amps to sum the signals and builds the second-order band-pass filter having a cut-off frequency in the range of 20–50 Hz. The total gain from input of the ADDER to the output of the band-pass filter is around 480. The output signal has an offset of 1.25 V and inverted because the ADDER is configured as negative feedback. The inversion can be easily flipped in the Matlab framework. The SUBTRACTOR uses the same op-amps for band-pass filter, but an instrumentation amplifier is used to build the SUBTRACTOR. The inputs of the SUBTRACTOR are coupled with a high-pass filter with a cut-off frequency of 20 Hz to bias the inputs and block the DC level from the sensors. The total gain is kept consistently with the ADDER which is around 480 for comparison. It also has the output offset at 1.25 V, but it is not in reversed form as the output of ADDER.

#### 2.3.3. Digital Signal Processing on 2 Accelerometers

The digital processing comprises two different combinations of data sets using two accelerometers in the Matlab framework. The signals from the two sensors placed in the same direction of all three axes go directly into the DAQ and their data are processed in two different strategies. Figure 5c shows the diagram of the digital processing. The three axes of the tri-axial accelerometer provide acceleration in three different planes. Therefore, the first strategy removes the motion noise by calculating the *TOTAL_ACC* of the heart vibration from all data of the 3 axes of the two accelerometers.

(1) $$TOTAL\_ACC=\sqrt{{\left(x1-x2\right)}^{2}+{\left(y1-y2\right)}^{2}+{\left(z1-z2\right)}^{2}}$$

where *x*1, *y*1, *z*1 are the data of three-axis of the first accelerometer and *x*2, *y*2, *z*2 are the data of three-axis of the second accelerometer. Then, the *TOTAL_ACC* is filtered with a digital zero-phase band-pass filter (20–50 Hz) for further noise removal. The filtered *TOTAL_ACC* is used for the detection step.

The second strategy uses only the combination of z-axis of both accelerometers; then, the combined data *(Z_ACC*) is also filtered with a digital zero-phase band-pass filter (20–50 Hz) for further noise removal. The filtered *Z_ACC* is used for the detection step.

(2) $$Z_{ACC}=abs\left(z1-z2\right)$$

where *abs* is the absolute function and *z*1, *z*2 are the z-axis data of two accelerators. The absolute function is used to ensure the difference of two z-axis data always positive so that there is no concern on the position interchange of the two accelerometers. The calculated acceleration is then passed through a FIR low-pass filter. The filter was designed by Matlab with the following configurations: 330 orders, 6 dB cut-off frequency at 6.7 Hz, 60 dB of stopband attenuation, and 1 dB of passband ripple. The parameters for filter design were obtained based on facts and experiments using Matlab to provide good performance and maintain characteristics of the SCG. A person must have systolic and diastolic ratio less than 1.2. The maximum heart beat is 220 beat-per-minute (bpm) and the minimum heart rate is 30 bpm. For the lowest heart rate, the systolic interval must be below 1091 ms. For the highest heart rate, the systolic period has to be under 149 ms. These set the frequency constraints. To maintain the timing and phase, all filters in the algorithm have the same order and are applied zero-phase filtering (filtering in both forward and reverse directions). As a result, it requires the number of input data of at least triple the order of the filter. For real-time detection, algorithm processes on segments of 1 s of data (i.e., 1000 samples) so that the order of all filters must be less than 333.

## 3. Results and Discussion

The results are compiled under the number of systoles and diastoles detected compared to the manual count from the reference ECG during testing. Moving average and interpolation methods were used to identify the systoles and diastoles. In the first method, the slope, a moving average threshold, and systolic interval constraint are utilized. In the second method, instead of using the threshold, the peak pattern of the SCG signal energy is used. The sample points of interpolation are the maxima of the SCG energy signal. Spline interpolation is applied in this algorithm. The collected ECG signal was used only for manual annotation and comparison. This moving average method has an average error rate of 4% for systolic detection and 9% for diastolic detection on the eight testing subjects. The average processing time of the moving average method is 75.2 ms for one-minute data which is suitable for realtime wearable devices in healthcare applications.

The results also present SNR and Vpp as indicators to compare the proposed method with existing methods. Both data of one-accelerometer and two-accelerometer method are filtered with the bandpass filter (20–50 Hz) using Labchart Reader software v8.1 to remove the mean. The Labchart reader was then used to calculate the variance of filtered data. The peaks of variance corresponding to systole and diastole were picked manually based on the ECG. The variance peak adjacent to the R peak of ECG is annotated as systolic variance. The variance peak next to the T wave of ECG is marked as diastolic variance. The variance in quiescent period (between a diastolic and consecutive systolic) was also picked manually. Then, the SNR for systole and diastole in each cycle are calculated by SNR_sys_ = Systolic variance/quiescent variance and SNR_dia_ = Diastolic variance/quiescent variance. The average of systolic and diastolic SNRs are calculated for all cycles. The Vpp of both systoles and diastoles are also picked manually cycle by cycle then calculate the average. The distortion level is also looked at. Signal-to-noise ratios of the systolic and diastolic phase are also calculated for comparison. 

### 3.1. Analog Processing on Two Accelerometers

#### 3.1.1. Using Subtractor

**Figure 6:**
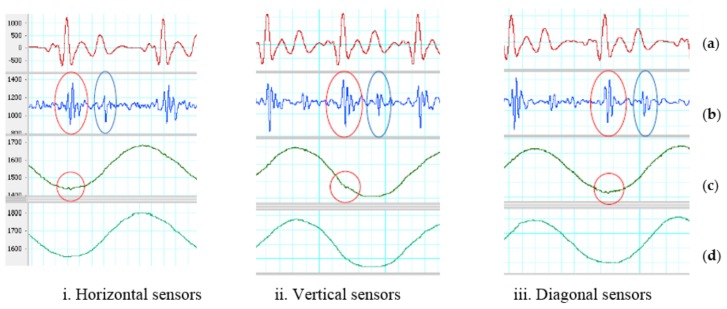
SUBTRACTOR on gentle movement. (**a**) Filtered ECG (5–20 Hz). (**b**) Filtered SCG (20–50 Hz). Red oval indicates an identified systolic. Blue oval indicates a recognized diastolic. (**c**) First sensor z-axis. Red circle indicates a small SCG signal. (**d**) Second sensor z-axis.

Under gentle motion, the two accelerometers placed horizontal, vertical and diagonal on the chest with SUBTRACTOR and the analog band-pass filter can effectively eliminate the artifact noise. All systolic and diastolic phases can be determined without the assistance from the reference ECG signal. Table 1 shows the average measurements on all recognized systoles and diastoles of the output signals on three types of sensor placement with SUBTRACTOR and gentle movement. The systolic Vpp of the output signals are about three times lower than using one accelerometer and the diastolic Vpp of the output signals are about two times smaller than using one accelerometer. The SNRs of horizontal and vertical placement alternate. The vertical sensors cope well with the noise on systolic portions, so the average systolic SNR of vertical placement is larger than the horizontal placement. On the other hand, the horizontal sensors cope better with the noise on diastolic portions, so the average diastolic SNR of horizontal placement is bigger than vertical placement. The diagonal placement handles the noise best on both systolic and diastolic portions that leads to high SNRs of both systoles and diastoles. The measured frequency of the gentle movement is around 0.9 Hz for all three sensor positions, so the analog band-pass can deal with it without any difficulty. The red and blue ovals in Figure 6 show determinable systole and diastole. There are also small SCG signals on z-axis of the sensor placed close to the heart which is indicated as red circles in the figure.

**Figure 7:**
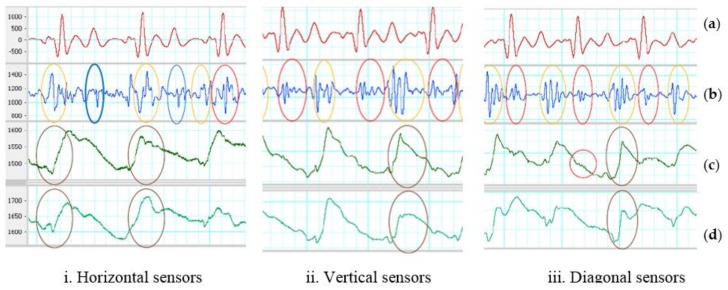
SUBTRACTOR on walking motion. (**a**) Filtered ECG (5–20 Hz). (**b**) Filtered SCG (20–50 Hz). Red oval indicates identified systoles. Blue oval indicates detected diastoles. (**c**) First sensor z-axis. Red circle indicates a small SCG signal, brown ovals show high-energy portion of walking. (**d**) Second sensor z-axis. Brown ovals indicate high-energy portion of walking.

Under walking motion, the two accelerometers placed horizontal, vertical and diagonal on the chest with SUBTRACTOR and the analog band-pass filter can deal with artifact noise better than using only one accelerometer. At least, no saturation is caused by walking motion even with the same high total gain when using one accelerometer. Although the SUBTRACTOR used high-performance common-mode rejection to suppress the walking noise and keep the output signal in range with the DAQ inputs, the two sensors do not react in the same way with walking motion which are shown by brown ovals in Figure 7. The results of different reaction are some distortion signals indicated by the orange ovals in the same figure. The difference of the two accelerometers may be due to the sensor discrepancy or sensor placement where both sensor axes are not laid on the same plane. The unaligned sensor placement can be observed by the dissimilar baseline values of the two z-axis signals. Another reason of different reaction can be each sensor placed on the half side of the chest creating different force-propagation path from each foot to each sensor when walking.

Table 2 shows the average measurements on all recognized systoles and diastoles of the output signals on three ways of sensor placement with SUBTRACTOR and walking motion. The SNR results may change due to how synchronized between high-energy portions of walking and systoles/diastoles. If they are in sync either with systoles or diastoles, those portions will be distorted and unidentified. If high-energy portions of the walking fall in the quiescent phase of output signals, both systoles and diastoles can be recognized with ECG reference. The average Vpp of distortion of horizontal placement is the smallest comparing to the other two. It means that the horizontal placement should handle the noise well which can increase the chance to determine cardiac phases. Two diagonal sensors do not remove the noise effectively because the average Vpp of distortion is quite high.

The z-axis of both sensors placed vertical on the chest looks quite similar comparing to the horizontal placement as shown in Figure 7. Besides the sensor discrepancy and sensor placement, two accelerometers placing on the same vertical line on the center of the chest may create almost identical force-propagation path from each foot to each sensor which results in similar response of both sensors. However, there still has different response from both vertical accelerometers which is depicted by the orange and brown ovals in the figure. The z-axis of both sensors placed diagonal on the chest look much different comparing to the horizontal placement. The causes may be the same with the horizontal and vertical placement which are the sensor discrepancies and sensor placements. Another reason can be the force-propagation paths are different on both vertical and horizontal directions comparing to only horizontal or vertical placement. One example of different response from both diagonal accelerometers which is illustrated by the orange and brown ovals in Figure 7. The red blue ovals show the determinable systoles and diastoles. There are also small SCG signals on z-axis of the sensor placed close to the heart which are indicated in red circles in Figure 7iii.

#### 3.1.2. Using Adder

**Figure 8:**
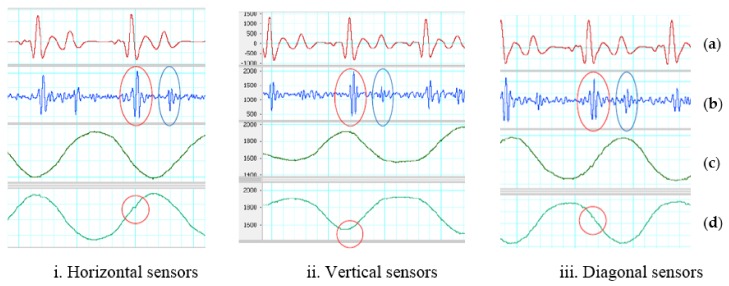
ADDER on gentle motion. (**a**) Filtered ECG (5–20 Hz). (**b**) Filtered SCG (20–50 Hz). Red oval indicates a recognized systole. Blue oval indicates an identified diastoles. (**c**) First sensor z-axis. (**d**) Second sensor z-axis. Red circle indicates a small SCG signal.

For gentle motion, the two accelerometers placed horizontal, vertical and diagonal on the chest with ADDER and the analog band-pass filter can remove most of the artifact noise. With or without the need of the reference ECG, all cardiac phases can be identified. The average measurements on all recognized systoles and diastoles of output signals on three types of sensor placement with ADDER and gentle movement are tabulated in Table 3. The systolic Vpp of the output signals in Figure 8 are around two times higher than using SUBTRACTOR and close to 1.9 V of the method using one accelerometer. Although the ADDER has high average Vpp on the output signals, their systolic and diastolic SNRs are not higher than the SUBTRACTOR for all three positions of the sensors. It means that the ADDER does not work well on motion noise comparing to the SUBTRACTOR. The addition enlarges not only the systolic and diastolic portions significantly but also the noise level at the output. The vertical sensors have the worst performance in the three positions. In addition, because of the sensor discrepancy, placing two accelerometers in the opposite directions for the ADDER can also make more difference in the response of the two accelerometers. Another reason of different reaction may be due to the dissimilar force-propagation path corresponding to sensor positions. The red and blue ovals show examples of determinable systoles and diastoles in Figure 8. There are also small SCG signals on the z-axis which are indicated as the red circles in the figure.

**Figure 9:**
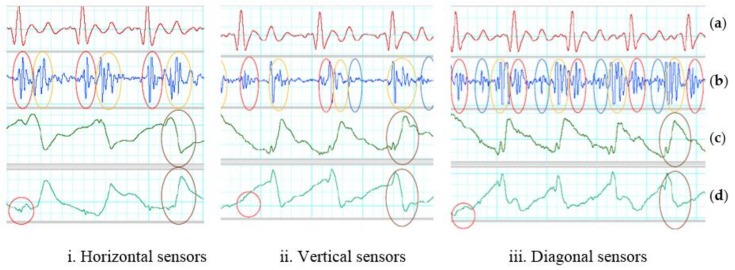
ADDER on walking motion. (**a**) Filtered ECG (5–20 Hz). (**b**) Inverted and filtered SCG (20–50 Hz). Red oval indicates recognized systoles. Blue oval indicates detected diastoles. Orange ovals show results of different reaction (**c**) First sensor z-axis. Brown ovals show high-energy portion of walking. (**d**) Second sensor z-axis. Brown ovals indicate a high-energy portion of walking. Red circle indicates a small SCG signal.

For walking motion, the two accelerometers placed horizontal, vertical and diagonal on the chest with ADDER and the analog band-pass filter cannot efficaciously tackle walking motion. Some saturation caused by walking motion appears on many portions of the output signals. Saturation also results in the loss of information and false solutions in the detection step. The possible cause is that the ADDER cannot eliminate totally the walking noise in high-frequency range, so the difference of the two z-axis signals is significant. The high inequality combining with high amplification gain leads to saturation on the output signal. In addition, sensor differences and the opposite direction of placement, the dis-similar force propagation path corresponding to sensor positions can be another reason of different response. Table 4 summarizes the performance of using two accelerometers with ADDER on walking motion. The output of both sensors placed horizontal in Figure 9 has least saturation portions comparing to the vertical and diagonal placements. Fortunately, the distorted portions of the horizontal placement do not exist all the time in the quiescence period, so its systolic and diastolic SNRs has higher values than the other two. However, the saturation dominates many systoles and diastoles, so only few of the phases are detected. The distorted portions of the vertical and diagonal placements unfortunately lay mainly in the quiescence period. Therefore, their SNRs are smaller than one even though almost systoles and diastoles are determined with the assistance of the ECG reference. The red and blue ovals illustrate examples of determinable systole and diastole in Figure 9. There are also small SCG signals on z-axis of the second sensor, and they are indicated as the red circles in the figure. The different responses on all horizontal, vertical and diagonal placements are depicted by the orange and brown ovals.

### 3.2. Digital Processing on Two Accelerometers

The two equations used in digital processing on the two accelerometers are repeated here for convenience:

(3) $$TOTAL\_ACC=\sqrt{{\left(x1-x2\right)}^{2}+{\left(y1-y2\right)}^{2}+{\left(z1-z2\right)}^{2}}$$

(4) $$Z_{ACC}=abs\left(z1-z2\right)$$

The following sub-sections comprises the results of using the total acceleration and z-axis acceleration to cope with gentle movement and walking motion in three places of the two sensors including horizontal, vertical, and diagonal. The gentle and walking data sets applied the above equations belong to analog processing with SUBTRACTOR because both sensors placing on the same directions for all axes have a better similarity on the reaction than placing on opposite directions when using ADDER. Hence, the results of both equations will also be compared to the results of analog processing with SUBTRACTOR.

For gentle movements, the process successfully eliminate artifact noises. All systolic and diastolic phases are identifiable referring to ECG signal, but the output signal of the SUBTRACTOR is lagged by 15 ms compared to the outputs of total acceleration and z-axis acceleration. The reason is the delay of the instrumentation amplifier and the band-pass filter in analog processing method. Table 5 shows the average measurements on all identified systoles and diastoles of the output signals including subtraction, total acceleration and z-axis acceleration on three types of sensor placement.

The average Vpp of systoles and diastoles of the subtraction is always greater than the total acceleration and z-axis acceleration, but its SNRs are always smaller than the total acceleration and z-axis acceleration. It means that the analog processing may also increase the output noise level through the process while digital processing suppresses the noise better at the output. Between total acceleration and z-axis acceleration, the average Vpp of systoles and diastoles are quite close to each other when placing the two sensors on horizontal and diagonal, but the SNRs of total acceleration are smaller than z-axis acceleration for horizontal placement while its SNRs are larger than z-axis acceleration for diagonal placement. The reasons for these are that the z-axis acceleration deal better with noise than total acceleration for horizontal placement and vice versa for diagonal placement.

**Figure 10:**
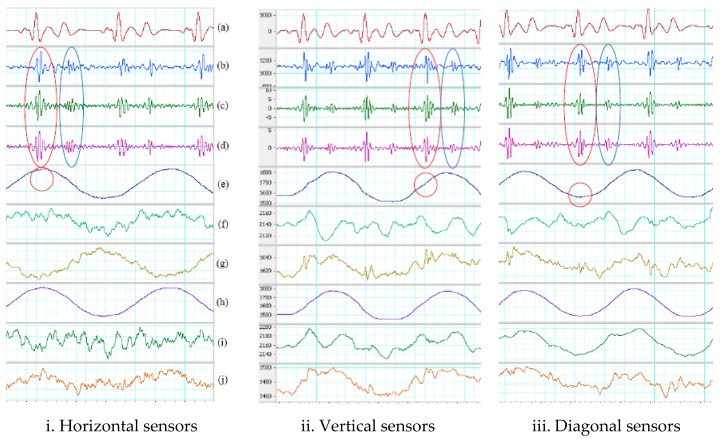
Digital processing on gentle movement. (**a**) Filtered ECG (5–20 Hz). (**b**) Filtered SCG with SUBTRACTOR (subtraction). (**c**) Filtered total acceleration. (**d**) Filtered z-axis acceleration. Red oval indicates a recognized systole. Blue oval indicates a determined diastole. (**e**) First sensor z-axis. Red circle indicates a small SCG signal. (**f**) First sensor y-axis. (**g**) First sensor x-axis. (**h**) Second sensor z-axis. (**i**) Second sensor y-axis. (**j**) Second sensor x-axis.

For vertical placement, the average Vpp of systoles and diastoles of total acceleration are greater than z-axis acceleration, but the SNRs of both total acceleration and z-axis acceleration are not much different. It means that although total acceleration may gather information of all three axes to achieve better Vpp, it can also accumulate the noise at output from all three axes rather than the noise only on one axis as z-axis acceleration. The red and blue ovals show examples of determinable systole and diastole in Figure 10. There are also small SCG signals on the z-axis of the first sensor indicated as red circles in the figure.

**Figure 11:**
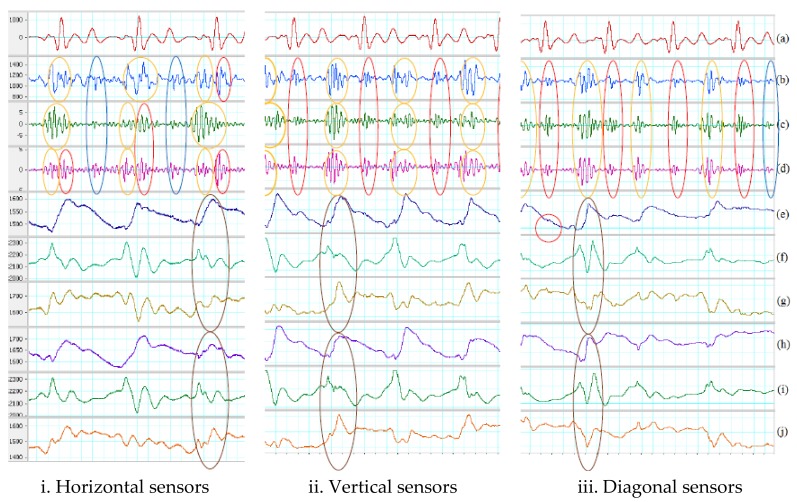
Digital processing on walking motion. (**a**) Filtered ECG (5–20 Hz). (**b**) Filtered SCG with SUBTRACTOR. (**c**) Filtered total acceleration. (**d**) Filtered z-axis acceleration. Red ovals indicate recognized systoles. Blue ovals indicate determined diastoles. Orange ovals show results of different response. Green ovals illustrate better noise suppression. (**e**) First sensor z-axis. Red circle indicates a small SCG signal. (**f**) First sensor y-axis. (**g**) First sensor x-axis. Brown oval indicates high-energy portion of walking. (**h**) Second sensor z-axis. (**i**) Second sensor y-axis. (**j**) Second sensor x-axis. Brown oval indicates high-energy portion of walking.

For walking motion, the process cannot totally get rid of the artifact noise on the data sets, but they suppress the noise better on some portions comparing to the SUBTRACTOR. Examples can be seen in the fifth cardiac period which are indicated by the green ovals in Figure 11. For the diagonal placement, outputs of the SUBTRACTOR and two equations are quite the same. It means that the two equations do not reduce much noise than the SUBTRACTOR in this position. Table 6 shows the average measurements on all recognized systoles and diastoles of the output signals using two equations of digital processing on walking data. Although the number of detected systoles and diastoles are mostly the same, the SNRs of both total acceleration and z-axis acceleration are virtually better than using the SUBTRACTOR only. The best results of SNRs belongs to z-axis acceleration on horizontal and vertical placements and total acceleration on diagonal placement. However, the SNR values of z-axis acceleration on horizontal and vertical placement are much greater than the total acceleration on diagonal placement. Hence, z-axis acceleration still has the best performance overall. The red and blue ovals show determinable systoles and diastoles in Figure 11. There are small SCG signals on the z-axis signal which are indicated by the red circles in the figure.

Table 7 and Table 8 summarize the results of the experiments on 8 subjects using the two different systolic and diastolic phase detection algorithms. For simplicity, only the results of the TOTAL_ACCELERATION and Z_AXIS are shown. The error rate is calculated by comparing with the collected synchronized ECG signal. Note that Subject 8 was not included in the statistic due to the abnormal data of this patient. The differences between the two methods in systolic and diastolic detection are not discussed in this article as the main focus of this work is in the implementation techniques to reduce motion artifacts.

## 4. Summary and Conclusions

**Figure 12:**
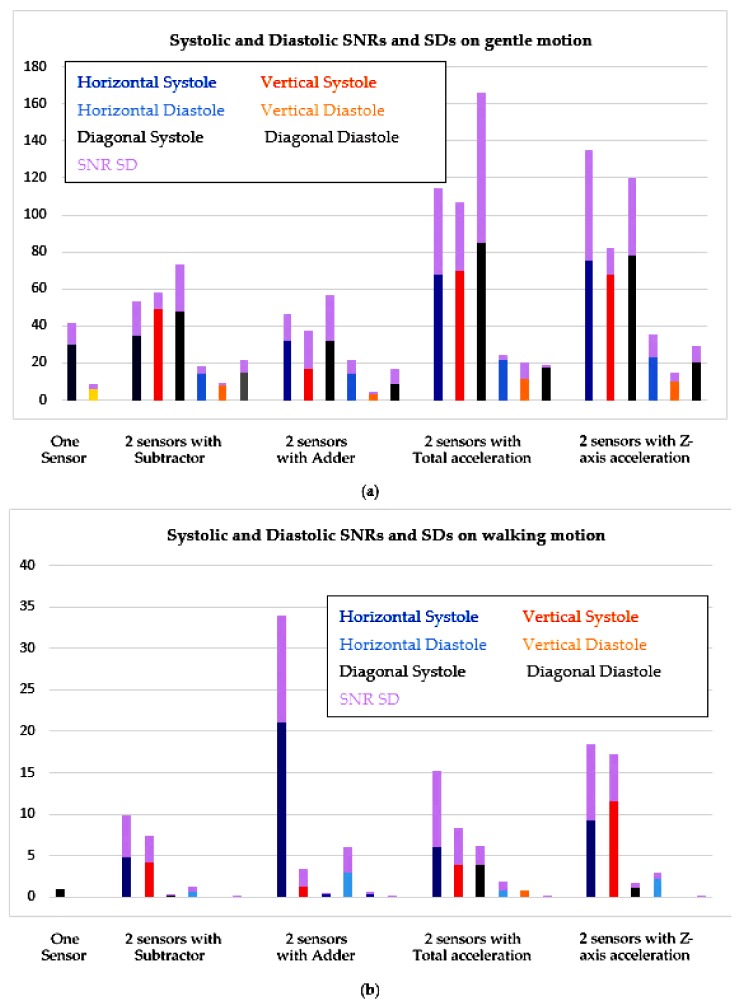
Comparison of SNRs on one sensor and two-sensor methods. (**a**) Gentle movement; (**b**) Walking.

A wireless DAQ combining with analog front-ends and algorithms on the Matlab framework are used to examine various motion removal techniques on the subjects while they were making soft movement or walking. There are two main procedures to remove the motion noise including analog processing and digital processing. They were tested on three placements of sensors including horizontal, vertical and diagonal with two kinds of motion noise comprising gentle movement and walking. Optimized bandwidth of the SCG signal was also analyzed on walking and running data of five subjects which is from 20 to 50 Hz. The noise removal techniques using two accelerometers provide better performance compared to traditional methods using only one sensor as summarized in Figure 12.

With gentle motion, almost all noise removal techniques can recover most systolic and diastolic portions of the SCG signal. The frequency of gentle motion is out of range of the band-pass filter, so it can be removed completely. Although the techniques of using ADDER have good Vpps on all three placements, the SNRs of total acceleration and z-axis acceleration are much better than the others on all placements. The total acceleration and z-axis acceleration improve average systolic SNR around 2 times and average diastolic SNR around 3 times comparing to using only one accelerometer. Because the performance is not significant different among sensor placements when coping with gentle motion, placing the sensor horizontally is the best as the sensor setup is very simple. With walking motion, all techniques cannot totally remove artifact noise due to walking. Although the Vpp of walking motion is smaller than the Vpp of gentle motion, the frequency of some walking portions still in the bandwidth of the bandpass filter, so they are amplified and overlap with the interested SCG signal. Again, the methods of using ADDER have good Vpps on all three placements and highest systolic SNRs on horizontal position. The SNRs of ADDER and z-axis acceleration are still mostly better than the others in all sensor positions. The SNRs of ADDER and z-axis acceleration are generally better than the others in all sensor positions; they enhance about 7 times of average systolic SNR and about 11 times of average diastolic SNR comparing to using one accelerometer. The performance of horizontal placement is outstanding comparing with other positions when coping with walking motion.

The total acceleration can enhance the SNRs with gentle motion by gathering all information from three axes, but with walking motion, the total acceleration may also accumulate the noise from three axes which causes lower SNRs comparing with ADDER and z-axis acceleration. The SNR results with walking motion may vary due to how synchronize between high-energy portions of walking motion with systoles/diastoles. If they are in-sync either with systoles or diastoles, those portions will be distorted and unidentified. If high-energy portions of walking stay in quiescent period of the output signals, both systoles and diastoles can be recognized. The reasons of less effectiveness of walking motion removal might be due to different reaction of two sensors to the motion. This can be the discrepancy or unaligned placement of the two sensors. The sensor reacts more similar with low-frequency noise such as gentle movement than high-frequency noise in walking, and the more the two sensor responses are different, the more the distortion appears on the output signal.

In this study, the combination of three axes of the two accelerometers using total acceleration was investigated and showed the enhancement in the results. In addition, the three positions of two sensors were tested to confirm which one can achieve the best quality SCG which has not been conducted by previous studies. The horizontal placement is simple, convenient, and provides better SCG waveforms. The dissimilar response from the two sensors may come from the un-aligned placement which means both sensors are not placed on the same plane. To overcome this challenge, a calibration procedure should be developed to compensate the different reaction of the two sensors. Based on detected systolic and diastolic phases from this proposed techniques and taking the advantages of the characteristics of the SCG signal (slopes, amplitudes, duration, etc.), the event-detection algorithm searches inside intervals to locate cardiac events. Moving average method or interpolation can be used to detect SCG events shown in Figure 1 from [2] and 6 new events identified in [25]. The results indicate the effectiveness and performance of the proposed multi-accelerometer concept. Furthermore, Matlab code can be converted to run in wearable device in real-time applications.

## Figures and Tables

**Table 1:** Signal measurements of using two sensors with SUBTRACTOR on gentle motion.

Sensor Placements	Systolic Vpp (mV)		Diastolic Vpp (mV)		Systolic SNR		Diastolic SNR	
	Average	SD	Average	SD	Average	SD	Average	SD
Two horizontal sensors	414.7	128.0	294.1	28.1	34.4	18.8	14.3	4.1
Two vertical sensors	418.2	33.0	162.8	20.2	49.5	8.8	8	1.3
Two diagonal sensors	539	112.1	281	64.6	48	25.1	14.9	7.1

**Table 2:** Signal measurements of using two sensors with SUBTRACTOR on walking motion.

Sensor Placements	Detected Systolic Vpp (mV)		Detected Diastolic Vpp (mV)		Distorted Vpp (mV)		Detected Systolic SNR		Detected Diastolic SNR	
	Average	SD	Average	SD	Average	SD	Average	SD	Average	SD
Two horizontal	524.4	21.2	272.8	49.7	427.2	110.8	4.8	5.1	0.7	0.5
Two vertical	368.6	29.5	N/A	N/A	699.3	55.8	4.2	3.2	N/A	N/A
Two diagonal	613.4	153.4	338	136.1	1478.7	495.4	0.2	0.1	0.07	0.1

**Table 3:** Signal measurements of using two sensors with ADDER on gentle motion.

Sensor Placements	Systolic Vpp (mV)		Diastolic Vpp (mV)		Systolic SNR		Diastolic SNR	
	Average	SD	Average	SD	Average	SD	Average	SD
Two horizontal sensors	1390.3	208.7	799.2	89.6	31.8	14.8	13.9	8.0
Two vertical sensors	1081.3	381.6	490.2	89.6	16.9	20.7	3.1	1.6
Two diagonal sensors	1600.9	321.4	850.1	183.6	32.2	24.4	8.6	8.1

**Table 4:** Signal measurements of using two sensors with ADDER on walking motion.

Sensor Placements	Detected Systolic Vpp (mV)		Detected Diastolic Vpp (mV)		Distorted Vpp (mV)		Detected Systolic SNR		Detected Diastolic SNR	
	Average	SD	Average	SD	Average	SD	Average	SD	Average	SD
Two horizontal	2324.8	120.6	1244.3	156.1	2176.4	11.0	21.1	12.8	3.0	3.0
Two vertical	1018.6	379.1	564.4	89.0	2366.6	34.1	1.3	2.1	0.3	0.4
Two diagonal	1836.1	124.6	1012.1	239.7	2385.3	76.2	0.4	0.03	0.1	0.03

**Table 5:** Signal measurements of two sensors with digital processing on gentle motion.

Sensor Placements and Methods		Systolic Vpp (mV)		Diastolic Vpp (mV)		Systolic SNR		Diastolic SNR	
		Average	SD	Average	SD	Average	SD	Average	SD
Two horizontal sensors	Subtraction	414.7	128.0	294.1	28.1	34.4	18.8	14.3	4.1
	Total acceleration	6.3	2.1	3.7	0.7	67.5	46.7	21.7	2.4
	Z-axis acceleration	6.2	2.3	3.6	0.8	75.3	60.1	23	12.7
Two vertical sensors	Subtraction	418.2	33.0	162.8	20.2	49.5	8.8	8	1.3
	Total acceleration	13.3	1.2	5.5	1.3	69.9	36.7	11.3	9.0
	Z-axis acceleration	6.6	0.6	2.4	0.3	67.8	14.4	9.7	5.0
Two diagonal sensors	Subtraction	539	112.1	281	64.6	48	25.1	14.9	7.1
	Total acceleration	11.7	4.6	4.7	1.0	84.7	81.2	17.8	1.4
	Z-axis acceleration	9.4	2.4	4.1	0.8	78.1	42.2	20.4	8.6

**Table 6:** Signal measurements of two sensors with digital processing on walking motion.

Sensor Placements and Methods		Detected Systolic Vpp (mV)		Detected Diastolic Vpp (mV)		Distorted Vpp (mV)		Detected Systolic SNR		Detected Diastolic SNR	
		Ave.	SD	Ave.	SD	Ave.	SD	Ave.	SD	Ave.	SD
Two horizontal	Subtraction	524.4	21.2	272.8	49.7	427.2	110.8	4.8	5.1	0.7	0.5
	Total acceleration	6.4	0.7	3.2	1.4	10.66	4.3	6	9.3	0.8	1.1
	Z-axis acceleration	6.9	1.0	3.5	0.6	4.1	1.1	9.3	9.1	2.2	0.7
Two vertical	Subtraction	368.6	29.5	N/A	N/A	699.3	55.8	4.2	3.2	N/A	N/A
	Total acceleration	10.3	3.4	5.09	N/A	12.4	8.7	3.9	4.5	0.8	N/A
	Z-axis acceleration	5.8	0.1	N/A	N/A	8.9	0.6	11.5	5.8	N/A	N/A
Two diagonal	Subtraction	613.4	153.4	338	136.1	1478.7	495.4	0.2	0.1	0.07	0.1
	Total acceleration	13.7	3.2	4.9	0.2	15.9	6.1	3.9	2.3	0.09	0.1
	Z-axis acceleration	10.1	2.0	4.4	0.4	20.7	7.4	1.1	0.7	0.04	0.04

Note: Ave. = Average

**Table 7:** The error and missing rates of moving average threshold and interpolation algorithms with total acceleration (Equation (3)) on eight subjects.

Sub-Jects	Manual				Moving Average				Interpolation			
	Heart Rate	Sys./Dias. Ratio	Total Sys.	Total Dias.	Systole (%)		Diastole (%)		Systole (%)		Diastole (%)	
					Error	Miss	Error	Miss	Error	Miss	Error	Miss
1	78.7 ± 2.7	0.50 ± 0.06	84	84	0	0	1.1	1.1	1.1	1.1	2.3	2.
2	88.7 ± 3.8	0.55 ± 0.09	90	90	2.2	3.3	2.2	3.3	1.1	3.3	1.1	3.3
3	75.2 ± 2.9	0.57 ± 0.09	76	77	1.3	0	1.3	1.3	2.6	1.3	2.6	2.6
4	98.9 ± 4.3	0.65 ± 0.10	100	100	0	0	5	5	0	1	1	2
5	76.4 ± 4.3	0.55 ± 0.04	80	79	0	0	0	0	1.2	2.5	1.2	1.2
6	79.7 ± 2.3	0.67 ± 0.10	84	84	0	0	1.1	1.1	0	2.3	1.2	3.5
7	70.6 ± 4.5	0.53 ± 0.12	72	73	6.9	4.1	9.5	8.2	5.5	4.1	8.2	8.2
8	102.0 ± 3.2	0.72 ± 0.11	105	106	3.8	58	3.7	58.4	13.3	25.7	13.2	26.4
Average	83.77	0.59	76.8	77.0	1.78	8.18	2.99	9.8	3.13.1	5.16	3.85	6.15
σ	11.52	0.07	11.4	11.4	2.5	20.2	3.1	19.8	4.5	8.4	4.5	8.4
Average without subject 8					1.2	1.1	2.4	2.7	1.2	2.3	2.2	3.5
σ without subject 8					2.6	2.1	3.6	3.4	3.1	1.5	4.0	2.9

Note: Sys. = Systolic, Dias.=Diastolic

**Table 8:** The error and missing rates of moving average threshold and interpolation algorithms with total Z-axis acceleration (Equation (4)) on eight subjects.

Subject	Manual				Moving Average				Interpolation			
	Heart Rate	Sys./Dia. Ratio	Total Sys.	Total Dias.	Systole (%)		Diastole (%)		Systole (%)		Diastole (%)	
					Error	Miss	Error	Miss	Error	Miss	Error	Miss
1	78.7 ± 2.7	0.50 ± 0.06	84	84	0	0	0	0	0	1.2	0	1.2
2	88.7 ± 3.8	0.55 ± 0.09	90	90	0	2.2	0	2.2	0	2.2	0	2.2
3	75.2 ± 2.9	0.57 ± 0.09	76	77	1.3	0	1.3	1.3	0	1.3	0	2.6
4	98.9 ± 4.3	0.65 ± 0.10	100	100	0	0	5	5	0	1	3	4
5	76.4 ± 4.3	0.55 ± 0.04	80	79	0	0	0	0	0	2.5	0	1.2
6	79.7 ± 2.3	0.67 ± 0.10	84	84	0	0	1.1	1.1	0	2.3	1.2	3.5
7	70.6 ± 4.5	0.53 ± 0.12	72	73	6.9	5.5	9.5	9.5	8.3	5.5	10.9	9.5
8	102.0 ± 3.2	0.72 ± 0.11	105	106	5.7	58	5.6	58.4	16.1	29.5	16	30.1
Average	83.77	0.59	76.8	77.0	1.74	8.21	2.81	9.69	3.1	5.69	3.89	6.79
σ	11.52	0.07	11.4	11.4	2.9	20.2	3.5	19.9	6.0	9.7	6.1	9.8
Average without subject 8					1.5	1.1	2.9	2.9	1.6	2.2	2.5	3.3
σ without subject 8					2.5	1.8	3.3	2.9	1.9	1.2	2.6	2.3

Note: Sys. = Systolic, Dias.=Diastolic
